# A Large Pericardial Effusion Culminating in Left Lung Collapse

**DOI:** 10.7759/cureus.5287

**Published:** 2019-07-31

**Authors:** Hina Amin, Bishal Gyawali, Debanik Chaudhuri

**Affiliations:** 1 Internal Medicine, State University of New York Upstate Medical University, Syracuse, USA; 2 Interventional Cardiology, State University of New York Upstate Medical University, Syracuse, USA

**Keywords:** lung collapse, pericardial effusion

## Abstract

Pericardial effusion is characterized by excess fluid accumulation in the pericardium. It can be asymptomatic or silent when the effusion is trivial in size or develops slowly. On the other hand, large rapidly developing effusions may present with hemodynamic instability or tamponade. In rare circumstances when a large effusion develops over a period of time, it may cause compression atelectasis of the surrounding bronchi and lung. We describe the case of a 70-year-old female who presented with acute respiratory insufficiency due to left lung collapse secondary to large pericardial effusion. To our knowledge, this is an extremely rare complication of large pericardial effusion.

## Introduction

Pericardial effusion is defined by the pathologic accumulation of fluid in the pericardium. The pericardial sac is fibro-elastic in nature hence accumulation of fluid leads to a rise in intra-pericardial pressure. Over a period of time, the pericardium stretches to accommodate the excess fluid and can cause compression atelectasis of the surrounding bronchi and lung. This leads to dullness on percussion of the left hemithorax below the tip of scapula and bronchial breathing on the exam, historically known as Ewart’s sign [1]. We aim to report a case of left lobar collapse secondary to extrinsic compression of the left main bronchus from a large pericardial effusion.

## Case presentation

A 70-year-old female with a medical history of diastolic heart failure, obstructive sleep apnea, and end-stage renal disease on hemodialysis, presented to our hospital with acute onset dyspnea. She did not have associated cough, sputum, fever, chest pain, or palpitation. Her blood pressure was 102/59 mm of Hg with a heart rate of 84 beats per minute. She was hypoxemic on ambient air with an oxygen saturation of 80%. Her oxygen saturation did not improve with supplemental oxygen, and therefore, she was placed on non-invasive positive pressure ventilation (NIPPV). Physical exam revealed distant heart sounds and diminished breath sounds with dullness over the left lung base. There was no jugular venous distension. Pulsus paradoxus was absent. Electrocardiogram showed sinus tachycardia with low voltage QRS complexes.

**Figure 1:**
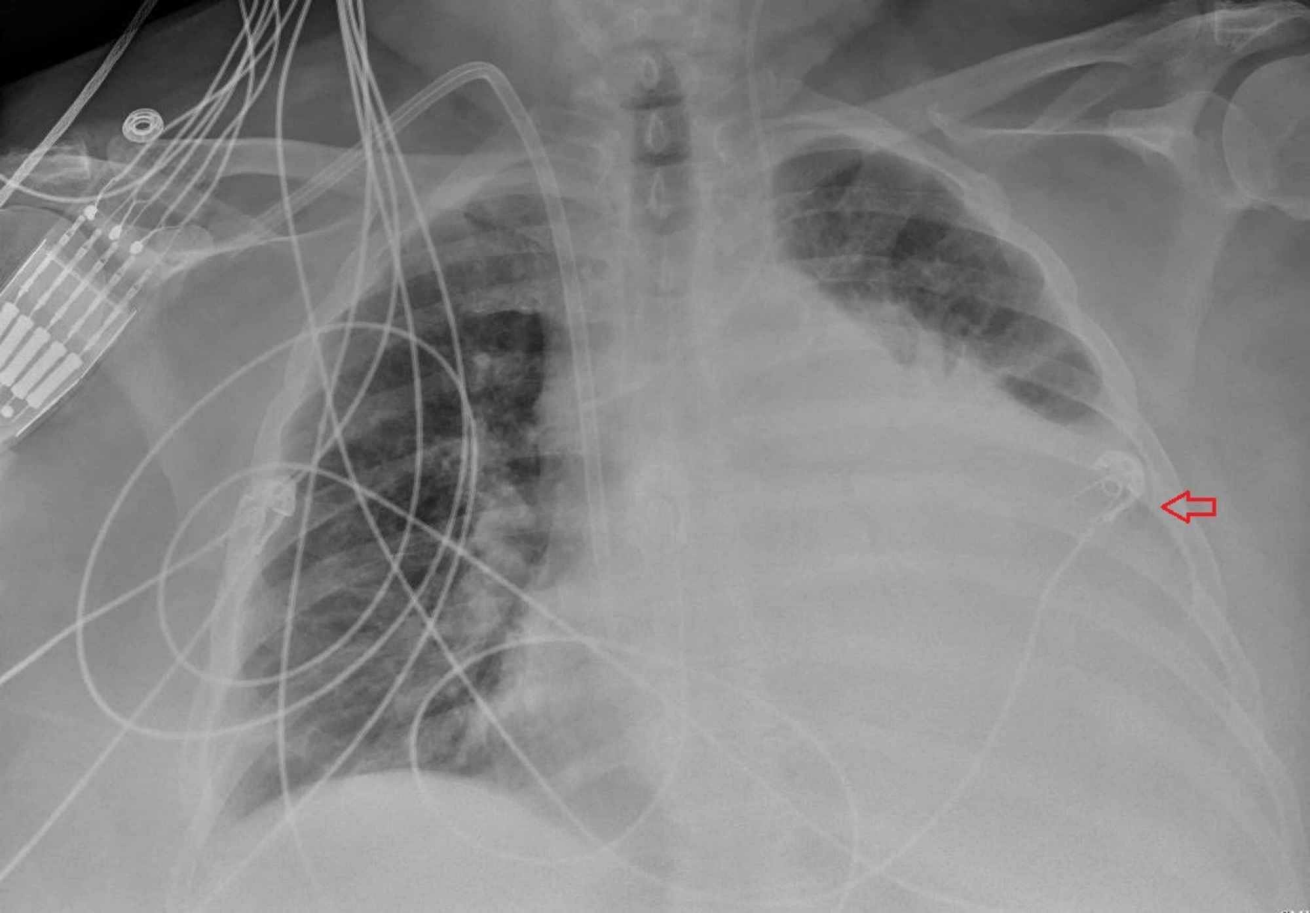
Chest X-ray showing enlargement of the cardiac silhouette and opacification of the left hemithorax (red arrow)

A chest X-ray was obtained and revealed marked enlargement of the cardiac silhouette concerning for pericardial effusion along with left lower lobe opacification suggestive of pleural effusion or atelectasis (Figure 1).

**Figure 2:**
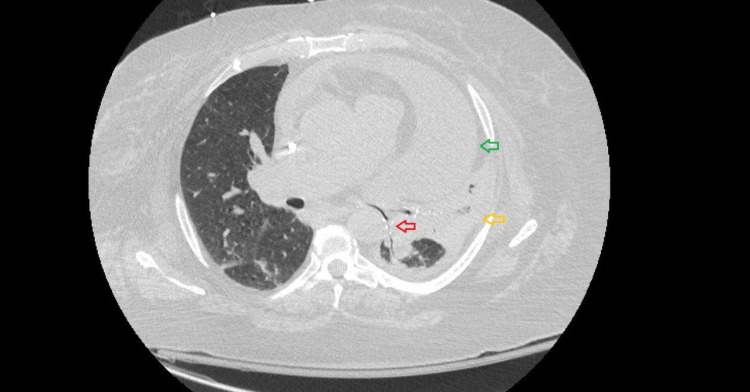
Computed tomography of thorax with a small left-sided pleural effusion (yellow arrow) and a large pericardial effusion (green arrow) compressing on the left bronchus and adjacent lung (red arrow)

A CT scan of the thorax further revealed compression of the left mainstem bronchus by the pericardial effusion resulting in atelectasis and collapse of the surrounding lung (Figure 2).

**Figure 3:**
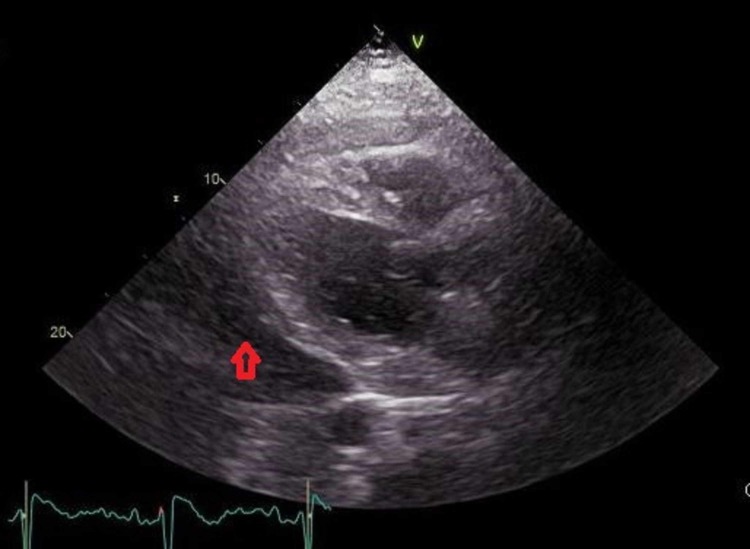
Transthoracic echocardiogram showed a large circumferential pericardial effusion (red arrow)

Transthoracic echocardiogram showed a large circumferential pericardial effusion without tamponade physiology (absence of right ventricle collapse and preservation of mitral velocities) (Figure 3).

**Figure 4:**
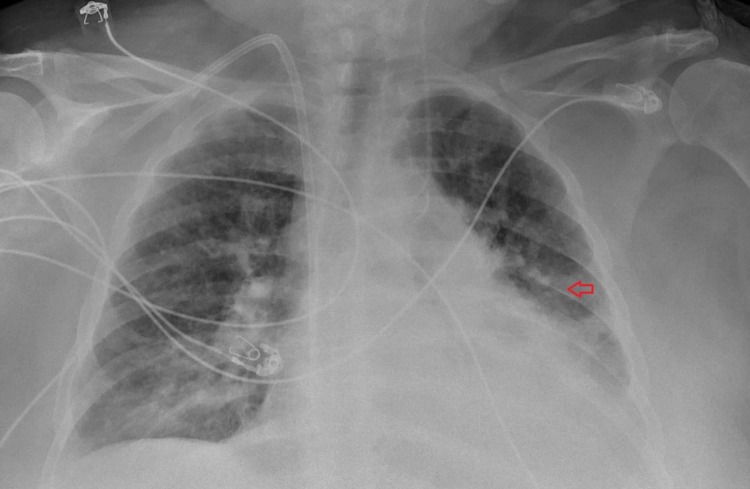
Repeat chest x-ray after pericardial drainage showing improved aeration of the lung (red arrow)

Given the absence of cardiac tamponade and potential for procedure-related complications, we decided to postpone pericardiocentesis. Instead, we directed our management towards symptom control and initiated dialysis to remove the fluid. Patient's symptoms initially appeared to improve, but a few days later, the patient had a recurrence of dyspnea along with hypoxemia at which point she needed to be placed on NIPPV again. We obtained a repeat transthoracic echocardiogram, which showed no interval change in the size of the pericardial effusion. Repeat chest X-ray revealed persistent opacification of the left hemithorax from lung collapse. At this time we performed a pericardiocentesis under ultrasound guidance removing 1.5 liters of blood-tinged fluid which relieved the compression of left bronchus and resolved associated dyspnea and hypoxemia. The fluid analysis revealed a total nucleated cell count of 3948/uL with differential count as follows: 3% epithelial lining cells, 5% lymphocytes, 16% neutrophils, 76% monocytes, and red blood cells (RBC) count of 110,787/uL. Fluid chemistry showed total protein of 4 g/dl, glucose 133 mg/dl, and lactate dehydrogenase (LDH) 1893 U/L. Follow up chest X-ray showed improved aeration of the lung (Figure 4). 

The patient’s respiratory distress improved significantly after pericardial drainage alone. She was able to maintain an oxygen saturation of >90% without positive pressure ventilation. A diagnostic left pleurocentesis was performed, and the fluid analysis revealed a total nucleated cell count of 677/uL with differential count as follows: 4% epithelial lining cells, 14% lymphocytes, 20% neutrophils, 62% monocytes, and RBC count of 80,500/uL. Fluid chemistry showed total protein of 3.1 g/dl, glucose 139 mg/dl and LDH 376 U/L, indicative of an exudative effusion. Both pleural and pericardial fluid cytology was negative for malignancy. We concluded that this patient's pericardial effusion was most likely from uremic pericarditis which also resulted in inflammation of the adjacent pleura.

## Discussion

The left mainstem bronchus in adults is approximately twice as long as the right and is horizontally positioned between the left pulmonary trunk anteriorly, descending thoracic aorta posteriorly, arch of aorta superiorly, and the left pulmonary veins and left atrial appendage inferiorly. By virtue of its location in the mediastinum, it is vulnerable to compression by surrounding structures. Lung collapse can result from either external compression or intrinsic obstruction of the bronchus. However, lung collapse from extrinsic compression of the bronchus from an enlarging pericardial effusion is rare in adults. This could be explained by the fact that normal adult bronchi, unlike in children, have a tough cartilaginous framework and hence are resistant to compression. A partial or complete collapse of the bronchus, as in this case, could signify an underlying defect in the bronchial cartilage or prolonged exposure to elevated pressure from the surrounding pericardial effusion. A literature search of MEDLINE/PubMed indexed cases yielded only two cases of pericardial effusion leading to left lung collapse which was successfully treated with pericardiocentesis [2]. The pericardial disease has also been associated with left pleural effusion which was observed in our case. Weiss et al. noted an isolated left-sided effusion in 21 of 35 patients with the pericardial disease and postulated permeability of parietal pericardium and extension of pericardial inflammation to the adjacent parietal pleura as possible mechanisms [3]. Treating the pericardial effusion and pericarditis leads to resolution of pleural effusion in a majority of cases.

## Conclusions

Lung collapse in the setting of pericardial effusion is a rare occurrence in adults. It should be suspected in patients with large pericardial effusions presenting with acute hypoxic respiratory failure and no evidence of cardiac tamponade. Clinicians should be aware of this rare presentation since the condition is largely reversible with pericardial drainage. 
